# Correlation between the accuracy of the emergency response centre’s urgency assessment and emergency medical services non-conveyance: a retrospective register-based study in Finland

**DOI:** 10.1186/s12873-024-01108-5

**Published:** 2024-10-15

**Authors:** Tomi Salminen, Kaius Kaartinen, Mira Palonen, Piritta Setälä, Eija Paavilainen, Sanna Hoppu

**Affiliations:** 1 Faculty of Social Sciences, Health Sciences Unit, Tampere University, Tampere, FI-33014 Finland; 2 Tampere University of Applied Sciences, Kuntokatu 3, Kuntokatu, FI-33520 Finland; 3 Centre for Prehospital Emergency Care, Tampere University Hospital, Wellbeing Services County of Pirkanmaa, Satakunnankatu 16, Tampere, FI-33100 Finland; 4 Nursing Research Foundation, Asemamiehenkatu 2, Helsinki, FI-00520 Finland; 5 Welfare Services County of Etelä-Pohjanmaa, Hanneksenrinne 7, Seinäjoki, FI-60220 Finland

**Keywords:** Ambulance, Emergency medical communication centre, Emergency medical dispatch, Emergency medical services

## Abstract

**Background:**

In modern emergency medical services (EMS), ambulances increasingly focus on examining and treating the patient at the scene. This has led to increased levels of non-conveyance. In Finland, for instance, approximately 40% of EMS dispatches end up in non-conveyance. As EMS systems evolve, the proportion of non-conveyance could serve as a cost-effective measure to assess the quality of the dispatch criteria, if a link to the performance of urgency assessment would be established. The purpose of this study was to investigate whether the proportion of non-conveyance is associated with the test performance levels of the urgency assessment. This investigation was done separately within each dispatch category.

**Methods:**

A retrospective evaluation of the data was conducted on all EMS dispatches in the Pirkanmaa Hospital District from 1 August 2021 through 31 August 2021. There were a total of 7,245 EMS dispatches during the study period of which 829 were excluded. This study was conducted by comparing the existing test performance levels (sensitivity, specificity and under- or overestimation) of the emergency response centre’s urgency assessment with the non-conveyance rate (%) of each dispatch category. The relationships between the variables were measured using Spearman’s rank correlation coefficient.

**Results:**

The proportion of over-triage was the only urgency assessment’s test performance variable that had a statistically significant correlation with the proportion of non-conveyance (*r* = 0.568; *p* = 0.003). Other test performance variables of the urgency assessment had no or little correlation to the proportion of non-conveyance. Of the 6,416 EMS dispatches in the study period, 42% (2,672) resulted in non-conveyance of the patient. In nine dispatch categories, at least half (51–69%) of the dispatches ended in non-conveyance.

**Conclusions:**

Based on this study, it seems that the percentage of non-conveyance in the dispatch category could be used, with certain limitations, to assess the proportion of over-triage in the dispatch category. The method is particularly applicable in scenarios where the dispatch criteria have undergone modifications and there is a need to monitor the effect of the changes on the level of over-triage.

**Supplementary Information:**

The online version contains supplementary material available at 10.1186/s12873-024-01108-5.

## Background

The historical role of ambulances was predominantly the conveyance of patients to the hospital. Currently, however, modern emergency medical services (EMS) may leave a patient without conveyance [1]. Presently, in most EMS systems, it is possible to examine and treat the patient at the scene, and thus, no conveyance is needed after treatment and assessment by paramedics [2]. Concurrently, people have a lower threshold to call the emergency number, which has led to a significant increase in the number of EMS dispatches in recent years [3]. The non-conveyance of patients who do not need immediate treatment is one way to respond to this increase in the burden on healthcare [4]. At the same time, the increase in the number of non-emergency dispatches has created the need to develop EMS units whose purpose is not to convey patients but rather to assess the need for help at the patient’s home [5].

In Finland, approximately 40% of EMS dispatches lead to the non-conveyance of a patient [6]. In most of these dispatches, a patient does not need any actual treatment but only a check-up and counselling, thus resulting in a significant unnecessary workload and costs for the EMS [7]. However, it is good to keep in mind that non-conveyance does not always describe the unnecessary use of EMS. There are situations in which it is necessary for the EMS to check the patient’s condition and rule out possible emergencies because laypersons do not have the ability and opportunity to assess the severity of the situation in the same way as paramedics [8, 9]. Various human reasons affect the perception of urgency by emergency callers. For instance, the patient’s age has been found to explain unnecessary EMS dispatches [10]. It is evident that caregivers of young children are more likely to be alarmed in response to seemingly benign symptoms, potentially leading to unwarranted emergency calls.

Factors related to non-conveyance have been studied in Finland and internationally [1–3, 11]. In previous studies, non-conveyance has also been used to measure the emergency response centre’s (ERC) urgency assessment [12]. However, to the best of our knowledge, studies on whether non-conveyance correlates with the accuracy of the urgency assessment made by ERC operators have not been carried out. The efficient and appropriate operation of the EMS requires a reliable and accurate urgency assessment from the ERC so that the dispatches can be carried out with appropriate resources [13]. The non-conveyance proportion of the dispatches would be a simple and cost-effective way to assess the quality of ERC operations if a relationship with the accuracy of urgency assessment is established.

The purpose of this study was to investigate whether the proportion of non-conveyance is associated with the test performance levels of the urgency assessment. This investigation was done separately within each dispatch category. Currently, the Finnish ERC Agency only uses the time of emergency call processing in the self-assessment of its operations [14]. There is no critical appraisal of the performance of the dispatch criteria provided by the EMS authority. One aim of the study was to obtain information on whether the non-conveyance proportion could serve as an indicator of the quality of the dispatch criteria.

## Methods

### Setting

In Finland, emergency calls from all authorities are handled by the ERC Agency, which forwards the dispatches to the responsible authorities [15]. In 2022, the six ERCs that exist in Finland received a total of 2,920,020 emergency calls, of which 861,120 were forwarded to EMS [14]. In Finland, emergency calls are handled by an ERC operator who is not a healthcare professional [16]. In the ERC’s assessment, EMS dispatches are divided into four urgency categories: A (suspicion of a life-threatening situation), B (urgent but stable situations), C (situations requiring urgent assessment) and D (non-urgent situations) according to the criteria provided by the EMS authority. Dispatches in the urgency categories A and B are handled as an emergency with lights and sirens and in categories C and D with normal driving. In addition, the reason for the emergency call determines the dispatch category [17]. After assessment and possible treatment at the scene, the EMS unit must always record either a conveyance code with a priority or a non-conveyance code. Conveyance code indicates the reason for the conveyance. Non-conveyance code indicates the reason why the patient was not conveyed.

There are two types of units in the Finnish EMS system: basic and advanced level. Basic level units are staffed with two at least basic-level paramedics who can be firefighters or nurses (registered or vocational). Advanced level units are staffed with at least one advanced level paramedic who can be nurse paramedic or registered nurse who is specialized in emergency care [18]. In the study area all EMS units are advanced level.

### Data

The test performance levels of the ERC’s urgency assessment (under- and overestimation rates, sensitivity and specificity) were previously determined according to dispatch categories by Salminen et al. [19]. We conducted a retrospective evaluation on the same data, consisting of all EMS dispatches in the Pirkanmaa Hospital District from 1 August 2021 through 31 August 2021. The data were previously gathered from the EMS management system, and they were supplemented with patient data (i.e., vital signs, treatment received from EMS and medical history) by research assistants. If the dispatch ended in non-conveyance, the EMS unit recorded the non-conveyance code into the EMS management system. Non-conveyance code indicates the categorical cause for the non-conveyance.

### Design and data analysis

This retrospective register-based study compared the existing test performance levels (under- and over-triage, sensitivity and specificity) of the ERC’s urgency assessment with the non-conveyance rate (%) of each dispatch category. All dispatch categories that had more than 50 dispatches during the study period were included. The proportions of non-conveyance were calculated based on non-conveyance codes by dispatch category. The test performance levels of the ERC’s emergency assessment by dispatch categories were previously published [19]. The proportion of non-conveyance, under- and over-triage, sensitivity and specificity were described by percentages.

The relationships between the variables were measured using Spearman’s rank correlation coefficient. The Spearman’s rank correlation coefficient was chosen as the method of analysis because most of the variables were skewed in distribution, except for the accuracy, which was normally distributed among the dispatch categories (*p* = 0.200).

## Results

**Fig. 1:**
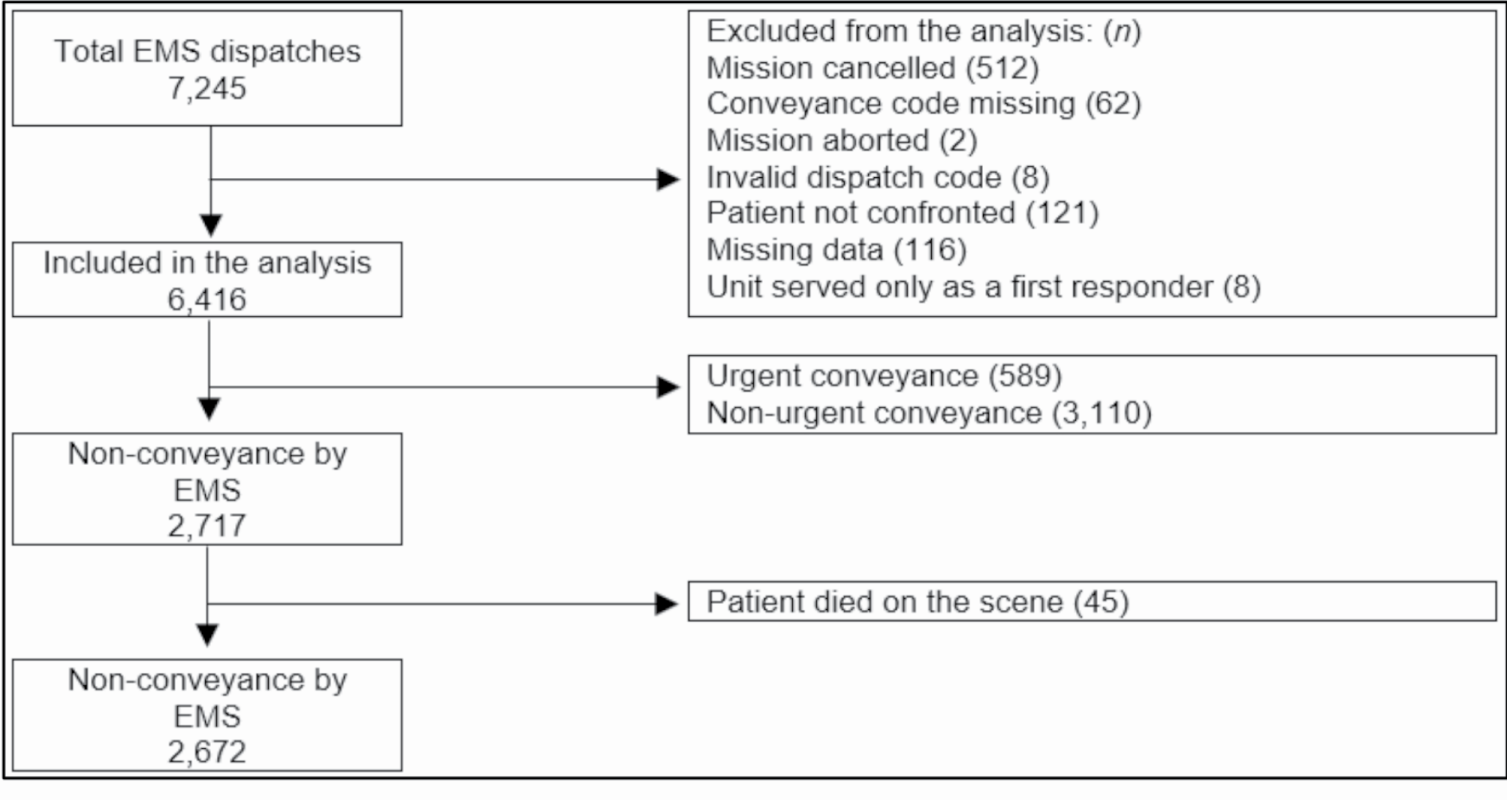
The data flow chart

During the study period, there were a total of 7,245 EMS dispatches, of which 829 were excluded. Of the 6,416 EMS dispatches approved, 2,717 ended in non-conveyance; 45 of the non-conveyances were due to the patient’s death at the scene and were therefore excluded from the analysis (Fig. 1). Also, the categorical causes of *Mission cancelled*, *Mission aborted*, and *Patient not confronted* were excluded, since the patient would not have been encountered in those cases.

Most common categorical cause for non-conveyance was *Patient’s state of health confirmed*,* no need for conveyance*. The distribution of the categorical causes of non-conveyance is shown in Table 1. Non-conveyance was most common in C urgency dispatches (46%) and lowest in A urgency dispatches (24%) the distribution according to dispatch urgencies is shown in Table 2.

**Table 1:** Categorical causes of non-conveyance

Patient’s state of health confirmed, no need for conveyance	1,510
Conveyance by other means	599
Patient treated on the scene, no need for conveyance	327
Patient refused treatment and/or conveyance	193
Patient died on the scene*	45
Patient taken into police custody	26
Other help received on the scene	17
Total:	2,717

*Not included in the analysis

**Table 2:** Dispatches and non-conveyances according to dispatch urgencies

	Dispatches	Non-conveyance
	*n*	n (*%*)
A urgency dispatch	341	83 (24)
B urgency dispatch	2,000	727 (36)
C urgency dispatch	2,260	1,039 (46)
D urgency dispatch	1,815	823 (45)
Total	6,416	2,672 (42)

All dispatch categories (*n* = 26) with more than 50 dispatches during the study period were included in the correlation review (see Additional file 1). These categories included a total of 6,153 (96%) dispatches. In nine dispatch categories, at least half (51–69%) of the dispatches ended in non-conveyance (Table 3). Non-conveyance was most common in the dispatch category *Traffic accident*,* small* (*n* = 67; 69%) and lowest in the dispatch category *Hospital transfer* (*n* = 5; 2%).

**Table 3:** Proportions of non-conveyance and urgency assessment test performance variables among the dispatch categories (%)

Dispatch category (*n*)	Non-conveyance	Over-triage^*^	Under-triage^*^	Sensitivity^*^	Specificity^*^
Traffic accident, small (97)	69	83	2.3	90	48
Allergic reaction (65)	66	80	0	100	36
Assault (54)	63	100	0	NA	93
Rhythm disorder (314)	62	81	0.9	89	77
Blood glucose problem (75)	57	68	5.4	67	80
Impact/hit (76)	55	80	0	100	45
Headache (110)	52	85	3.1	78	61
Limb pain (144)	51	78	0.7	67	95
Body pain (59)	51	100	7.3	0	93
Cut, wound (67)	49	70	0	100	73
Chest pain (631)	49	80	4.3	95	21
Unspecific symptoms ^+^(75)	45	88	NA	100	0
Abdominal pain (286)	44	68	2.0	69	91
Poisoning (224)	43	50	8.6	78	75
General weakness (984)	43	81	2.1	59	88
Back pain (183)	43	78	1.7	40	96
Breathing difficulty (407)	42	72	4.5	84	61
Traffic accident, bicycle etc. (109)	40	68	0	100	68
Psychiatric symptom ^+^(325)	39	NA	5.8	0	100
Fall (882)	37	79	1.9	71	85
Convulsion (137)	33	64	3.8	94	48
Stroke (306)	31	74	3.6	97	23
Nausea, diarrhoea, constipation (140)	31	33	2.2	40	99
Unconscious ^+^(99)	30	58	NA	100	0
Cardiac arrest (51)	4	8.3	0	100	43
Hospital transfer (253)	2	30	12.0	84	76

NA: not available. ^+^There was only A/B or C/D dispatch priority available, and this inhibited the calculation of some variables. *Test performance variables (Over-triage, Under-triage, Sensitivity, and Specificity) by Salminen et al. 2023 [19].

The proportion of over-triage was the only urgency assessment’s test performance variable that had a correlation with the proportion of non-conveyance (Table 4). As the proportion of over-triage increased, so did the proportion of non-conveyance (*r* = 0.568; *p* = 0.003). Other test performance variables of the urgency assessment had no or poor correlation to the proportion of non-conveyance.

**Table 4:** Correlations between non-conveyance and urgency assessment’s test performance variables

	Proportion of non-conveyance	
	*r*	*p*
Proportion of over-triage	.568^1^	0.003*
Proportion of under-triage	‒0.213^1^	0.318
Proportion of sensitivity	‒0.008^1^	0.970
Proportion of specificity	0.039^1^	0.852

^1^Spearman’s rank correlation coefficient. *p < 0.05.

## Discussion

According to our results, non-conveyance appeared to have a correlation with the proportion of the ERC’s urgency over-triage. Although overestimating urgency and non-conveyance does not mean the same thing, there was a strong correlation between them. This means that it could be possible to use non-conveyance as an indicator in the evaluation of dispatch criteria performance. Non-conveyance could serve as one of the indicators when assessing the share of over-triage, which is a key performance measure for efficient ERC operations [13].

If the share of non-conveyance is used in the assessment of ERC operations, a few confounding factors must be considered. One important factor that affects the percentage of non-conveyance, in addition to the patient’s condition, is the patient’s will. As in many other Western countries, in Finland, patients have the right to decide on their treatment, and a conveyance to a hospital against the patient’s will requires certain precise criteria to be met and a doctor’s request for assistance by the police [20]. Thus, the patient can refuse treatment, as well as conveyance, even if the patient’s condition is serious. Thus, the dispatch can be accurate, even though it ends in non-conveyance. Some other causes for non-conveyance, such as the patient not being confronted, the mission being cancelled or the patient dying, should also be ignored if the non-conveyance is to be used as a measure of over-triage in urgency assessment. This is technically easy to implement in Finland, as the categorical causes for non-conveyance exist in the EMS management system as their own codes.

Another factor to consider is the specific features of certain dispatch categories. A good example of this is *Blood sugar imbalance*, in which there is a relatively low percentage of over-triage of urgency when compared with the proportion of non-conveyance. This is most likely due to the nature of the symptoms in question. Low blood sugar is a life-threatening condition for a diabetic, but it can be easily treated on the scene. This leads to non-conveyance, even though it was originally an emergency. Therefore, consideration is needed for each dispatch category if a non-conveyance proportion is used as a measure of the over-triage rate, as it may also contain appropriate dispatches. This issue becomes irrelevant if the non-conveyance rate is used to monitor changes in the proportions within the dispatch category. For example, it might prove useful when assessing the impact of changes made to the dispatch criteria. It is also crucial to note that no substantial correlation was found between under-triage and non-conveyance. Thus, the low non-conveyance rate did not seem to be associated with the increased risk of under-triage.

These study results indicate that non-conveyance of the patient was quite common in general. More than a third of dispatches resulted in non-conveyance in most analysed categories. Of all dispatches, 42% culminated in non-conveyance, which aligns closely with prior studies in Finland [3, 7]. Internationally, the rate of non-conveyance varies greatly (3.7–93.4%) between different regions and studies [11]. This variability most probably reflects the diversity of different systems and practices in different regions.

Non-conveyance was less common with more severe symptoms, such as resuscitation and unconsciousness. A notable exception is the category *Vomiting*,* nausea and diarrhoea*, which stands out as the only non-critical symptom among the six lowest rates of non-conveyance. Analysing the proportion of non-conveyance across different urgencies revealed a trend where the percentage of non-conveyance tended to increase as urgency decreased, although there was little difference between C and D urgency, which aligns with a prior study in Finland [2]. There was almost double non-conveyance in the C urgency as in the A urgency (24% vs. 46%). This indicates that the urgency categories managed to describe the patient’s condition, even though there was a high proportion of non-conveyance in all urgencies. This result was significantly different from a Swedish study, where the non-conveyance proportion was almost the same in all urgency categories [21].

It is crucial to recognise that non-conveyance does not mean that the patient will not require EMS care; it only indicates that one does not need conveyance by ambulance. The patient may have received significant or even lifesaving help at the scene. Additionally, it is important to remember that non-conveyance is a complex human decision made with limited means of assessment [22]. A computer-aided risk assessment has been proposed to support decision-making [23]. There are risks associated with non-conveyance, and approximately 5% of patients required assistance due to an adverse event that occurred after non-conveyance. Therefore, it is inappropriate to directly interpret that the patients who were not conveyed did not need hospitalisation. The patient’s age, gender, ethnicity and poverty are factors that have previously been found to increase the risk of re-alerting the EMS [1, 24].

### Strengths and limitations

The strength of this study was the ability to exclude certain categorical causes of non-conveyance. The use of a total proportion of non-conveyance would have led to increased bias because of non-conveyance causes that do not reflect the urgency of the situation (such as *Mission cancelled*, *Mission aborted*, or *Patient not confronted*). That is why it might not be possible to use non-conveyance proportion as an indicator of over-triage in EMS systems where it is not possible to screen non-conveyances according to categorical causes. This was a local study, and if the results are to be utilised, differences between EMS systems must be considered. Additionally, sample size, use of a non-parametric test, a limited period of data collection time and observational design must also be taken into account.

## Conclusions

Non-conveyance could be used as a cost-effective indicator to express the number of inappropriate EMS dispatches but not entirely unambiguously. Based on this study, it can be concluded that the percentage of non-conveyance in the dispatch category could be used, with certain limitations, to assess the proportion of over-triage in the dispatch category and further studies are needed. The method is particularly applicable in scenarios where the dispatch criteria have undergone modifications and there is a need to monitor the effect of the changes on the level of over-triage. In such cases, the distinctions and unique characteristics among dispatch categories do not matter as comparisons are conducted within a single dispatch category. Use of any tools that could improve the appropriateness of the urgency assessment carried out by the ERC must be considered, as they would very likely reduce unnecessary EMS dispatches.

## Electronic supplementary material

Below is the link to the electronic supplementary material.

Supplementary Material 1: Numbers and proportions of non-conveyance and urgency assessment test performance variables among the dispatch categories.

## Data Availability

All data generated or analysed during this study are included in this published article [and Additional file 1.pdf].

## Abbreviations

EMS: Emergency medical services
ERC: Emergency response centre
