# Supplementary material for: Correlation between the accuracy of the emergency response centre’s urgency assessment and emergency medical services non-conveyance: a retrospective register-based study in Finland
Source: BMC Emerg Med. 2024 Oct 15;24:193. doi: 10.1186/s12873-024-01108-5 (PMC11481283; doi:10.1186/s12873-024-01108-5)
Supplement: Supplementary file 1 — Supplementary Material 1: Numbers and proportions of non-conveyance and urgency assessment test performance variables among the dispatch categories. [file 12873_2024_1108_MOESM1_ESM.pdf]

Additional file 1. Numbers and proportions of non-conveyance and urgency assessment test performance variables among the dispatch categories.

Dispatch category (*n*)	Non-conveyance *n*	Non-conveyance %	Over-triage %	Under-triage %	Sensitivity %	Specificity %
Traffic accident, small (97)	67	69,0	83,0	2,3	90,0	48,0
Allergic reaction (65)	43	66,0	80,0	0,0	100,0	36,0
Assault (54)	34	63,0	100,0	0,0	NA	93,0
Rhythm disorder (314)	194	62,0	81,0	0,9	89,0	77,0
Blood glucose problem (75)	43	57,0	68,0	5,4	67,0	80,0
Impact/hit (76)	42	55,0	80,0	0,0	100,0	45,0
Headache (110)	57	52,0	85,0	3,1	78,0	61,0
Limb pain (144)	74	51,0	78,0	0,7	67,0	95,0
Body pain (59)	30	51,0	100,0	7,3	0,0	93,0
Cut, wound (67)	33	49,0	70,0	0,0	100,0	73,0
Chest pain (631)	309	49,0	80,0	4,3	95,0	21,0
Unspecific symptoms +(75)	34	45,0	88,0	NA	100,0	0,0
Abdominal pain (286)	125	44,0	68,0	2,0	69,0	91,0
Poisoning (224)	96	43,0	50,0	8,6	78,0	75,0
General weakness (984)	420	43,0	81,0	2,1	59,0	88,0
Back pain (183)	78	43,0	78,0	1,7	40,0	96,0
Breathing difficulty (407)	171	42,0	72,0	4,5	84,0	61,0
Traffic accident, bicycle etc. (109)	44	40,0	68,0	0,0	100,0	68,0
Psychiatric symptom +(325)	126	39,0	NA	5,8	0,0	100,0
Fall (882)	327	37,0	79,0	1,9	71,0	85,0
Convulsion (137)	45	33,0	64,0	3,8	94,0	48,0
Stroke (306)	96	31,0	74,0	3,6	97,0	23,0
Nausea, diarrhoea, constipation (140)	43	31,0	33,0	2,2	40,0	99,0
Unconscious +(99)	30	30,0	58,0	NA	100,0	0,0
Cardiac arrest (51)	2	4,0	8,3	0,0	100,0	43,0
Hospital transfer (253)	5	2,0	30,0	12,0	84,0	76,0

NA: not available

+There was only A/B or C/D dispatch priority available, and this inhibited the calculation of some variables.
